# A case study for teaching information literacy skills

**DOI:** 10.1186/1472-6920-9-7

**Published:** 2009-01-29

**Authors:** Karla V Kingsley, Karl Kingsley

**Affiliations:** 1 College of Education, Teacher Education Department, University of New Mexico, Albuquerque, NM, USA; 2 Department of Biomedical Sciences, School of Dental Medicine, University of Nevada, Las Vegas, Las Vegas, NV, USA

## Abstract

**Background:**

The Internet has changed contemporary workplace skills, resulting in a need for proficiency with specific digital, online and web-based technologies within the fields of medicine, dentistry and public health. Although younger students, generally under 30 years of age, may appear inherently comfortable with the use of technology-intensive environments and digital or online search methods, competence in information literacy among these students may be lacking.

**Methods:**

This project involved the design and assessment of a research-based assignment to help first-year, graduate-level health science students to develop and integrate information literacy skills with clinical relevance.

**Results:**

One cohort of dental students (n = 78) was evaluated for this project and the results demonstrate that although all students were able to provide the correct response from the content-specific, or technology-independent, portion of the assignment, more than half (54%) were unable to demonstrate competence with a web-based, technology-dependent section of this assignment. No correlation was found between any demographic variable measured (gender, age, or race).

**Conclusion:**

More evidence is emerging that demonstrates the need for developing curricula that integrates new knowledge and current evidence-based practices and technologies, traditionally isolated from graduate and health-care curricula, that can enhance biomedical and clinical training for students. This study provides evidence, critical for the evaluation of new practices, which can promote and facilitate the integration of information literacy into the curriculum.

## Background

Use of the Internet has changed contemporary American life in ways that were unimaginable two decades ago. Proficiency with digital technology and online communications are crucial skill-based methodologies for conducting evidence-based research in all realms, including the fields of medicine, public health, and higher education. On a typical day almost half (49%) of Internet users search for information related to their work, leisure activities, education, and/or health care, according to research conducted by the Pew Internet and American Life Project [1]. In the six-year period between 2002 and 2008, the percentage of users who searched for general information on the Web climbed by 69%; during the same period, the number of Internet users who searched specifically for information about health-related topics surged by 79% [2]. As everyday life becomes increasingly digitized, Internet users face new challenges as they endeavor to solve information problems.

Without a doubt, in-depth knowledge about subject matter, theory, and pedagogy are vital components of contemporary college and university-level teaching. In order to ensure the effective integration of information and communications technologies (ICT) into teaching, faculty also need an adequate understanding of and proficiency with ICT [3,4]; however, this is not always the case. Researchers at the Pew Internet & American Life project noted a substantial generation gap between college professors and their students with regard to Internet usage, interests, and abilities [5]. A measure of reluctance among university faculty regarding the use of web-based technology in the classroom remains, and as a result, researchers continue to underscore the need for professional development for educators at the university level [4,6,7].

University students may appear to be more comfortable in technology-intensive environments than are their professors, but it does not necessarily follow that they have the knowledge and critical thinking skills to effectively locate, filter, and evaluate information found online. A core competency for operating in electronic environments is information literacy; however, until recently information literacy initiatives were primarily the concern of librarians [8]. A national survey conducted by the Pew Internet & American Life project, entitled *Information searches that solve problems*, found that 63% of those who used the Internet were successful in finding the information they needed, but only 57% of users seeking information specifically about health-related matters were successful [9].

Although support for evidence-based medicine (EBM) has grown in recent years, as means of improving patient outcomes as well as improving the overall quality and effectiveness of healthcare delivery, case studies assessing clinically integrated EBM courses incorporating ICT in the form of digital technologies and online web searches are less abundant [10,11]. Recent studies have found that methods for teaching EBM are not only inconsistent among medical and dental schools, but may also be underdeveloped, suggesting a general lack of consensus regarding which methods represent best educational practice [11,12]. Although a variety of methods exist for both teaching and learning of EBM skills, it is becoming increasingly clear that these methods should incorporate substantial components of ICT, e-learning and must include guidance to acquire the skills for filtering and establishing the quality of current information gathered during this process [10,12].

These data demonstrate the need to integrate information literacy skills (ILS), specifically using web-based technologies that students will likely use in clinical practice following graduation. This study describes the development and dissemination of a research-based assignment, integrating web-based technologies to acquire theoretical and applied knowledge and concepts of a dental curriculum, within a specific first-year dental course. In addition, assessment of student performance, as well as recommendations for future modifications, are presented to provide a focused, targeted assignment with the potential to be adapted and implemented in a variety of teaching and learning environments.

## Methods

### Course

Current dental students (n = 78) enrolled in a first year dental course, DEN7110: Oral Pathogens and Oral Immunology, during Spring 2008 were given an assignment designed to help them develop and integrate information literacy skills with clinical relevance. In brief, this first year (DS1) course is designed to build a foundational knowledge base of oral pathogens and immunity, to help students describe the impact of oral pathogens on the orofacial areas, the concepts of mucosal immune mechanisms and the pathogenic mechanisms of the oral flora. Dr. Kingsley is a lecturer in this course.

### Assignment Description

This assignment was designed with three specific objectives and outcomes in mind:

1. Describe the scientific basis of a caries vaccine and provide an example of its application in patients. (Upon completion of this exercise, the student will be able to discuss biomedical science concepts of caries immunology and caries vaccines in the context of oral health and disease);

2. Compile a bibliography of eight (8) articles that represent the current literature in the area of caries microbiology and virulence factors (3) and caries vaccines (5) in refereed journals (The student will be able to critically evaluate relevant primary scientific literature regarding caries immunology and caries vaccines using and integrating web-based technologies, such as PubMed);

3. From the articles in this bibliography, provide an analysis of the two articles that are considered the "best" evidence and defend the selection of each one. (The student will be able to build and review an updated bibliography of current literature regarding caries vaccines)

In brief, students were given a review article of vaccines against caries (dental or tooth cavity formation) from 2001 and were then asked to provide answers related to content (technology-independent) and also to use specific web-based, online technologies to find more recently published peer-reviewed citations (technology-dependent) [see Additional file 1].

### Assessment and Evaluation

The assignment consisted of four questions designed to gauge students' content knowledge in various aspects of caries immunology and caries vaccines. Three questions, which addressed separate aspects of fundamental knowledge, were divided into two parts, A and B. Part A of each question was content specific; obtaining full credit was based solely upon the students' ability to list or define the correct response(s). Part B of each question involved technology or web-based technology to search for citations and building relevant literacy skills; obtaining full credit was based upon the students' ability comprehend the overall task, translate this knowledge into a new context and apply this knowledge in a new, specific situation, evidenced by the citation. Parts A and B were scored separately, as correct or incorrect and responses tallied.

### Human Subjects Exemption

Student assessment data for this assignment were retrieved and each record was assigned a numerical, non-duplicated identifier to prevent the disclosure, and ensure the confidentiality, of personally identifiable private information. Gender, age and race were noted separately for each student record, in separate tables, prior to assignment.

This protocol was reviewed by the UNLV Biomedical Institutional Research Board (IRB), and was deemed excluded from IRB review (OPRS#0811-2911). Informed Consent was waived pursuant to the exemption to human subjects research under the Basic HHS Policy for Protection of Human Research Subjects, (46.101) Subpart A (b) regarding IRB Exemption for 2) research involving the use of education tests (cognitive, diagnostic, aptitude, achievement) where the subjects cannot be identified or linked, directly or through identifiers, to the individual subjects.

## Results

**Figure 1:**
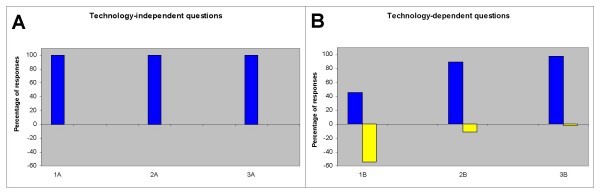
**First year (DS1) student assignment evaluations**. A) Student responses to technology-independent questions revealed all students reported correct responses to content-specific sections (1A, 2A and 3A). B) Student responses to technology-dependent questions revealed only 54% of students reported incorrect responses to 1B, with fewer incorrect responses to 2B and 2C (11 and 2%, respectively) (blue = correct, yellow = incorrect).

The DS1 student assignment evaluations were provided in non-identifiable format to the study authors, revealing only the number of responses and percent of correct responses for each of four questions, part A and B (n = 624). Analysis of this assignment revealed that virtually all students (n = 78) had sufficiently demonstrated their knowledge of major ideas, relating to the content-specific or technology-independent portions of questions 1–3 (Part A), however many students demonstrated lack of proficiency with information literacy and the technology-dependent application of skills (Part B) (Figure 1).

Specifically, 100% of students had correct responses to the content-specific or technology-independent portion of questions 1, 2 and 3 (Part A): 1A, 2A and 3A (Fig. 1A). Fewer than half (46%) provided correct responses to the information literacy or technology-dependent portions of question 1 (Part B), although a significantly greater proportion of students had correct responses to question 2 and question 3 (89% and 98%, respectively). Responses were scored as incorrect if the source was not found evidence-based and available through PubMed, as stated explicitly in the instructions (Appendix 1), with most incorrect responses citing websites and not peer-reviewed sources. Half of students providing incorrect responses to 2B also provided an incorrect/incomplete response to question 1B.

**Figure 2:**
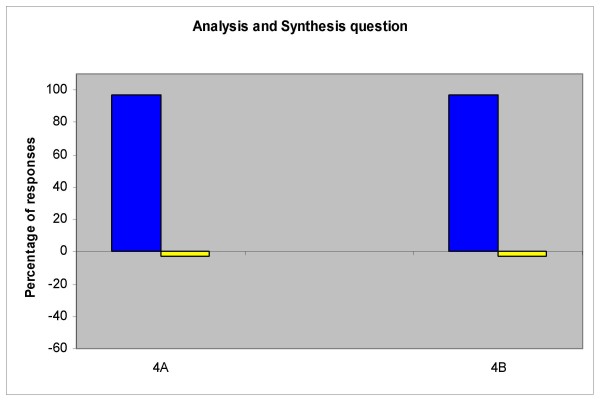
**First year (DS1) responses to analysis and synthesis question**. Nearly all students were able to provide correct responses to the technology-dependent (4A) and technology-independent (4B) sections of this question (97%).

Student responses to question 4, the analysis and synthesis of information portion, were also separated into technology-dependent (4A) and technology-independent (4B) sections (Figure 2). Nearly all students (97%) were able to provide appropriate citations utilizing the web-based interface (4A), with the same proportion (97%) demonstrating their ability to analyze and summarize data obtained from one of these sources (4B). Interestingly, neither of these two students that provided an incorrect response to 4A had missed any other previous technology-dependent question.

To determine if other characteristics or demographic factors may have affected student performance, age, gender and race for incorrect responses were compared with the cohort averages (Table 1). Although age did not appear to be a significant factor, it should be noted that the average age was under 30 and did not vary significantly within the cohort (24.9 +/- 2.2 years). A slightly higher proportion of males missed one or more of the technology-dependent questions (Part B) 82%, compared with the cohort average of 76%. A somewhat higher proportion of non-white students also missed one or more of the technology-dependent questions (Part B) 25%, compared with the cohort average of 19%. Neither of these differences were statistically significant (p > 0.05).

**Table 1:** Characteristics of respondents

**Demographic**	**Cohort**	**Incorrect respondents**
Age	Ave = 24.9 years, STD = 2.2	Ave = 24.8 years, STD = 2.7 21.8% (51/234)

Gender	Male = 76% (59/78); Female = 24% (19/78)	Male = 82% (42/51); Female = 18% (19/51)

Race	White = 81% (63/78); Other = 19% (15/78)	White = 75% (38/51); Other = 25% (13/51)

## Discussion

In this study we examined graduate students' ability to utilize web-based technologies as an integral part of the research process. In order to complete the assignment, students made use of several different technology-dependent skills: the ability to locate online library resources, as well as an understanding of how information is organized within the library system, how to access online databases, and how to interpret and evaluate research materials within the context of a specific discipline. The current study adds to the small but rapidly growing corpus of research specifically focused on university students' levels of information literacy.

As web-based technologies grow more prevalent in the digital era, so too does the need for students to acquire and fine-tune their 21^st^-century skills, including their information finding abilities. As previously stated, a national survey conducted by the Pew Internet & American Life project found that 63% of those who used the Internet were successful in finding the information they needed, and only 57% of users seeking information related to health-related information [9]. As the results of Q1 Part B from this case study clearly demonstrated, fewer than half of graduate-level health science students were able to demonstrate competence on the first web-based, technology-dependent assignment. Because no standardized methods yet exist for both teaching and learning of EBM skills, it is imperative that health science and dental curricula, should incorporate substantial components of ICT, e-learning and specific guidance for acquiring the skills for filtering and establishing the quality of current information from the evidence base during this process.

It is also interesting to note that analysis of the cohort using demographic variables, limited in this study to age, gender and race, did not provide any evidence that these results were correlated with any particular group – although the group composition was overwhelmingly young, white and male. Some research has suggested that age and income are the factors most responsible for the "digital divide" [13], while more recent studies indicate that race and gender differences are the primary factors that predict information literacy skills and associated academic performance measures [14]. Because the digital divide appears to increase with each ten year age bracket (60+ > 50–59 > 40–49, etc.) it is not surprising that no correlation was found in this study, in part because there were no significant differences between the age of those who responded incorrectly (24.8 y/o) and the average age of all students in the cohort (24.9 y/o), and also because of the relative age of this particular cohort does not approach the ages where significant differences in ICT and ILS were previously found. This study also did not find any significant differences based on race or gender, although a higher proportion of those scoring incorrectly were minorities (25%), compared with their overall representation in the cohort (19%), suggesting that studies including a larger number of students, as well as a greater proportion of females and minorities, may find significant differences.

Additional factors, representing potential confounders of the previously mentioned studies [13,14], include the role of income and its association with prior educational experience before entering dental school. Although access to these specific data for students in this cohort was not available for the study authors, summary data exists and has been released for this cohort that may be relevant to the present study. The Office of Admissions released statistics that demonstrate 11.5% (9/78) of this cohort had no four year degree, 8.9% (7/78) attended undergraduate institutions that offered no masters- or doctoral-level programs and 76.9% (60/78) attended public institutions of higher learning – all potential influences of the undergraduate education experience in gaining information retrieval and literacy skills. For example, many capstone or senior-level courses are cross-listed with masters- or doctoral-level courses that tend to expand critical thinking skills, foster student-student teaching and learning, and may reinforce evidence-based learning [15,16]. Because these courses are more likely to take place during the final or senior year and in masters- or doctoral-granting institutions, those students who enter dental school without completing their undergraduate degree, often citing financial reasons, may be more likely to miss these learning opportunities. Finally, although the role of English as a second language (ESL) may represent one additional difficulty facing students and ICT and ILS, only one student was listed as having graduated from a non-US institution, and no further data regarding ESL for this cohort was available.

## Conclusion

These results strongly suggest the need for designing and incorporating information literacy and integration of technology-dependent, applied research assignments into graduate-level curricula. Although some evidence exists for guidance on successful evaluation strategies during the process of developing information literacy skills [17,18], relatively few examples of specific courses and specific recommendations can be found [19]. The results of this study suggest that placement of ILS and ICT teaching and learning modules should be integrated and incorporated early in the graduate curriculum. Furthermore these results also demonstrate cause for concern, considering that levels of information literacy can either enhance, or constrain, students' ability to complete technology-dependent assignments or conduct research, which are increasingly common skills needed for everyday clinical practice [20].

The nature and extent of technology-enhanced pedagogy and curricula are also directly tied to levels of information literacy on the part of educators. While there is a significant body of literature that discusses technology integration in schools and classrooms at all levels of education, more research is needed that specifically addresses the issue of information literacy, particularly with regard to university-level learners, and even more specifically – integration of technology and web-based applications in dental, medical and health science settings to prepare clinicians for the demands of 21^st ^century practice [21-23].

During the process of analyzing and presenting these data, several areas for future research were identified, which may have significant potential as the subject of future research endeavors and studies. Since the ultimate goal is to provide teaching and learning opportunities related to information retrieval skills in the context of evidence-based practices, two foci have been identified as having higher priority. First is the identification of additional first-year dental courses in other introductory clinical, pre-clinical and behavioral science courses within the curriculum that can facilitate similar integration of modules and assignments into student coursework. Second is the incorporation of multiple searching strategies and bibliographic databases in order to providing expanding learning opportunities, to provide comparisons and contrasts, as well as facilitating more detailed feedback that could be used to improve the effectiveness of the information retrieval process and subsequent evaluation by students.

## Competing interests

The authors declare that they have no competing interests.

## Authors' contributions

KK and KVK conceived and coordinated this project and were equal contributors. KK administered the assessment and KVK assisted with the interpretation and analysis of data generated and made significant contributions to the writing and editing of this manuscript.

## Pre-publication history

The pre-publication history for this paper can be accessed here:



## Supplementary Material

Additional file 1 **Caries vaccine student assignment.** Four part student assignment with technology-dependent and technology-independent questions given for first year dental students. Click here for file
