# Supplementary material for: A case study for teaching information literacy skills
Source: BMC Med Educ. 2009 Jan 29;9:7. doi: 10.1186/1472-6920-9-7 (PMC2640392; doi:10.1186/1472-6920-9-7)
Supplement: Additional file 1 — Caries vaccine student assignment. Four part student assignment with technology-dependent and technology-independent questions given for first year dental students. [file 1472-6920-9-7-S1.doc]

Caries Immunology and Vaccine

Research Assignment

DEN7110: Oral Pathogens and Oral Immunology

Spring 2008

Summary:

Dental caries is among the most prevalent diseases affecting human populations. Homeostatic changes of the normal oral bacterial ecology, and an overgrowth of specific bacteria, such as *Streptococcus mutans*, are the primary causal factors associated with the formation of caries lesions. Most dental treatments target the elimination of this caries-causing bacterium – although more recent strategies have been designed to prevent the colonization of *S. mutans* and other bacterium through vaccination.

Assignment:

Given the following review article, Michalek, Katz and Childers A Vaccine against Dental Caries. *BioDrugs* (2001) 15 (8): 501-508, summarize briefly your answer to the following questions (1-3):

1. Is dental caries infectious? Provide at least one citation

2. Which oral microorganisms are associated with caries? Provide at least one citation

3. What are the virulence factors of these organisms? Provide at least one citation/each

4. Given that this article is almost seven years old, provide an updated bibliography

Instructions:

Go to PubMed at the following URL: <http://www.ncbi.nlm.nih.gov/sites/entrez>

Use appropriate search terms (i.e. caries, vaccines, immunology)

1. **Find and list at least five (5) articles** specific to caries vaccine design or development that were published more recently than Michalek, Katz and Childers (2001).
2. For one (1) of these articles, briefly summarize the vaccination strategy (active, passive), the immunogen (adhesin, etc.) and the results (did it work?).

Objectives and Outcomes:

1. **Describe the scientific basis of a caries vaccine and provide an example of its application in patients.** (Upon completion of this exercise, the student will be able to discuss biomedical science concepts of caries immunology and caries vaccines in the context of oral health and disease);
2. **Compile a bibliography of eight (8) articles that represent the current literature in the are of caries microbiology and virulence factors (3) and caries vaccines (5) in refereed journals**. (The student will be able to critically evaluate relevant primary scientific literature regarding caries immunology and caries vaccines using and integrating web-based technologies, such as PubMed);
3. From the articles in this bibliography, provide an anlysis of the two articles that are considered the “best” evidence and defend the selection of each one. (The student will be able to build and review an updated bibliography of current literature regarding caries vaccines)
